# Cobblestoned ankles in Graves disease: koebnerized elephantiasic pretibial myxoedema

**DOI:** 10.1093/skinhd/vzag019

**Published:** 2026-04-16

**Authors:** Mohammed Shanshal, Georgios Kallikas

**Affiliations:** Department of Dermatology, Imperial College Healthcare NHS Trust, London, UK; Faculty of Medicine, Imperial College London, London, UK; Department of Histopathology, Imperial College Healthcare NHS Trust, London, UK

## Abstract

A 61-year-old man with Graves disease presented with bilateral perimalleolar cobblestoned plaques, elephantiasic pretibial myxoedema, with a linear koebnerized tract. Biopsy confirmed thyroid dermopathy (compact orthokeratosis, acanthosis, abundant interstitial dermal mucin on Alcian blue–PAS). This vignette spotlights the dermatology–internal medicine interface: a distinctive cutaneous sign of thyroid autoimmunity with atypical ankle/foot involvement and trauma/friction as a cofactor.

Dear Editor, A 61-year-old Afro-Caribbean man with autoimmune thyroid disease (post-thyroidectomy) and active Graves orbitopathy had a 5-year history of slowly progressive thickening and presented with bilateral, left-greater-than-right perimalleolar verruciform (‘cobblestoned’) plaques on xerotic skin. A linear koebnerized tract extended proximally from the left plaque (Figure 1a, b). There was no ulceration or clinical history of chronic venous or lymphatic insufficiency. At presentation, thyroid tests showed thyroid stimulating hormone (TSH) 0.26 mIU L^–1^ (low; reference range 0.30–4.20), free T4 14.8 pmol L^–1^ (within range; reference range 9.0–23.0) and TSH receptor antibody >30 U L^–1^ (positive; lab reference <0.4). Biopsy showed compact orthokeratosis, acanthosis, verrucous epidermal hyperplasia and abundant interstitial dermal mucin separating collagen bundles, highlighted by Alcian blue–periodic acid–Schiff, with scant inflammation: findings diagnostic of pretibial myxoedema (PTM) (Figure 1c, d). Pathogenesis reflects TSH receptor antibody-driven fibroblast activation with dermal/subcutaneous hyaluronan accumulation.^1^

**Figure 1:**
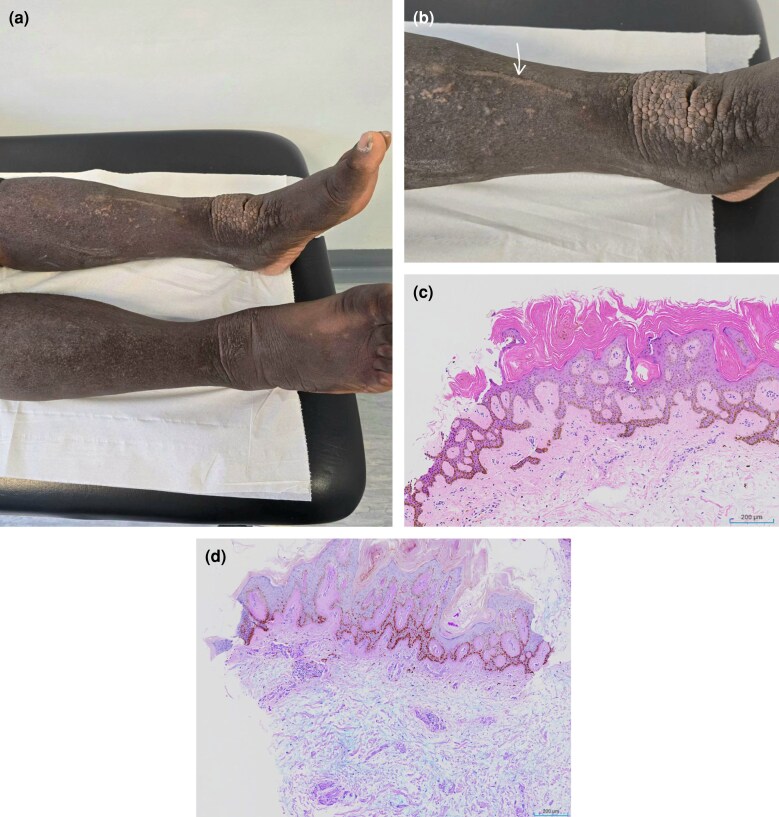
Clinical and histopathological features of elephantiasic pretibial myxoedema. (a) Bilateral perimalleolar, cobblestoned plaques (left > right). (b) Close-up of the left ankle showing coalescent papules and a linear koebnerized tract (arrow). (c) Histology (haematoxylin and eosin stain): verruciform epidermal hyperplasia overlying a myxoid dermis with splayed collagen bundles and scant inflammation. (d) Histology (Alcian blue–periodic acid–Schiff stain): abundant interstitial dermal mucin (blue) separating collagen bundles.

The elephantiasic (cobblestoned) variant is among the rarest described subtypes of thyroid dermopathy (≈1–3% across cohorts).^1,2^ PTM can involve sites beyond the shins, including ankles and feet and other pressure-prone locations.^2^ The linear koebnerized tract in our patient supports the contribution of local trauma/friction to lesion development and distribution.^2,3^ Management centres on superpotent topical corticosteroids under occlusion (often with compression); for bulky or refractory plaques, intralesional corticosteroids are a supported treatment option,^1^ and illustrative successful use has been reported in elephantiasic disease.^4^ Responses are variable, and tend to be poorer in elephantiasic disease,^1^ yet our patient achieved meaningful improvement with a moderate-potency topical corticosteroid alone.

## Data Availability

Not applicable.
